# Case Report: Digital fabrication of a music splint oral appliance for lip irritation in a brass player

**DOI:** 10.3389/fpsyg.2026.1854538

**Published:** 2026-10-05

**Authors:** Mana Ando, Mariko Hattori, Yuichi Yamatani, Satoshi Obata, Takashi Mori, Hiroshi Churei, Akira Nishiyama, Gen Tanabe, Yuka Sumita, Noriyuki Wakabayashi

**Affiliations:** 1 Department of Advanced Prosthodontics, Institute of Science Tokyo, Tokyo, Japan; 2 Dental Laboratory, Institute of Science Tokyo Hospital, Tokyo, Japan; 3 Yamaha Corporation, Hamamatsu, Japan; 4 Department of Masticatory Function and Health Science, Institute of Science Tokyo, Tokyo, Japan; 5 Department of General Dentistry, Institute of Science Tokyo, Tokyo, Japan; 6 Department of Sports Dentistry, Meikai University School of Dentistry, Sakado, Japan; 7 Department of Partial and Complete Denture, The Nippon Dental University, Tokyo, Japan

**Keywords:** case report, digital dentistry, embouchure, lip pain, prosthodontics, wind instrument player

## Abstract

Playing a wind instrument requires coordinated orofacial function, and oral conditions may influence both comfort and musical performance. Among brass musicians, dental crowding or irregular incisal morphology may cause mechanical irritation of the lip while playing. This case report describes the fabrication and evaluation of a music splint for a professional trombonist with recurrent lower-lip pain while playing. A conventionally fabricated provisional splint was first provided, followed by a definitive splint designed using a digital workflow based on intraoral scanning and computer-aided design. Subjective outcomes were evaluated using a visual analog scale, and objective outcomes were assessed by psychoacoustic and rhythm analyses under multiple performance conditions. Both the provisional and the definitive splints reduced lip pain and improved playing comfort. After adjustment of the definitive splint, the expected dynamic patterns of loudness and sharpness were maintained, while rhythm stability was similar to that observed without a splint, suggesting no marked deterioration in the measured performance characteristics. This case suggests that digitally fabricated music splints may be a useful clinical option for managing lip irritation in brass musicians while preserving musical performance.

## 1 Introduction

The orofacial region plays an essential role in singing and musical performance (Czech and Alt, 2024). When playing a wind instrument, oral structures such as the lips, tongue, and teeth are closely involved in forming and controlling the embouchure (Watson and Price, 2020; Woldendorp et al., 2016). The direction and magnitude of forces applied to the reed or mouthpiece vary depending on the instrument, and these force patterns are associated with dental alignment, tooth position, and the condition of the lips and jaw (Porter, 1967; Herman, 1974; Woldendorp et al., 2016). Clemente et al. (2019) demonstrated that the angle between the mouth and the instrument differs across wind instruments, suggesting that the force distribution on the dentition and the mechanical load on the lips also vary. These biomechanical differences may influence embouchure stability and playing sensation, indicating that oral conditions are likely to affect musical performance in wind instrumentalists.

Accordingly, several studies have explored the relationship between wind instrument performance and oral conditions. Experimental and pilot studies have found that changes in oral morphology, such as the placement of a palatal appliance, can alter performance characteristics, including pitch range and dynamic control (Hattori et al., 2014). In addition, a systematic review suggests that tooth position and occlusal conditions may influence embouchure comfort and playing ability (van der Weijden et al., 2018). However, the current evidence remains limited because of small sample sizes, the wide variety of instruments studied, and inconsistent evaluation methods. Therefore, the clinical significance of dental factors in wind instrument performance has not yet been clearly established, particularly with respect to the effects of dental interventions on musical performance.

Among the oral conditions relevant to wind musicians, lip pain and inflammation are clinically important concerns (Czech and Alt, 2024). Brass players may experience excessive mechanical irritation of the lips during performance due to dental crowding, protrusion, or sharp incisal edges, which can lead to discomfort, inflammation, and pain. Such symptoms may reduce endurance and impair tone control (Storms et al., 2016). Contact between the mouthpiece and the mandibular anterior teeth has also been associated with mucosal erosion of the lower lip (van der Weijden et al., 2018). To address these issues, intraoral devices such as music splints have been introduced to protect the lips from pressure between the teeth and the instrument, and previous reports suggest that these devices may reduce pain with minimal acoustic impact (Tanabe et al., 2022).

At the same time, advances in digital technology have improved the precision of dental appliance design and fabrication (Nii et al., 2023). Digital workflows are increasingly used in dentistry, and their application in care for musicians is gaining attention (Hattori et al., 2026). However, reports describing digitally fabricated intraoral devices specifically for wind instrumentalists remain limited. Therefore, the present case report describes a trombonist in whom a definitive music splint was fabricated using digital technology based on a conventionally manufactured provisional appliance.

## 2 Case description

### 2.1 Patient information and clinical findings

A 36-year-old male professional trombonist presented to our clinic with a chief complaint of lip pain while playing. The patient reported that he had been concerned about the alignment of his mandibular anterior teeth for approximately 4 years. During this period, he also experienced discomfort and pain in the lower lip while playing. The symptoms became more pronounced during prolonged playing sessions, interfering with endurance and causing early fatigue.

The patient had started playing the trombone in junior high school and had continued daily practice since then. At the time of presentation, he was actively working as a professional musician, performing in a jazz band and teaching as an instructor. He had no significant medical or dental history.

As shown in Figure 1A, intraoral examination revealed crowding of the mandibular anterior teeth, a crossbite on the left side, and an edge-to-edge relationship of the right lateral incisors. No ulceration of the lower lip was observed at the time of examination; however, the patient reported recurrent irritation in the same area of the lower lip following extended performance. Despite the presence of these occlusal characteristics, stable occlusal contacts were confirmed using articulating paper in maximum intercuspation. No signs or symptoms of temporomandibular disorders were observed.

**Figure 1:**
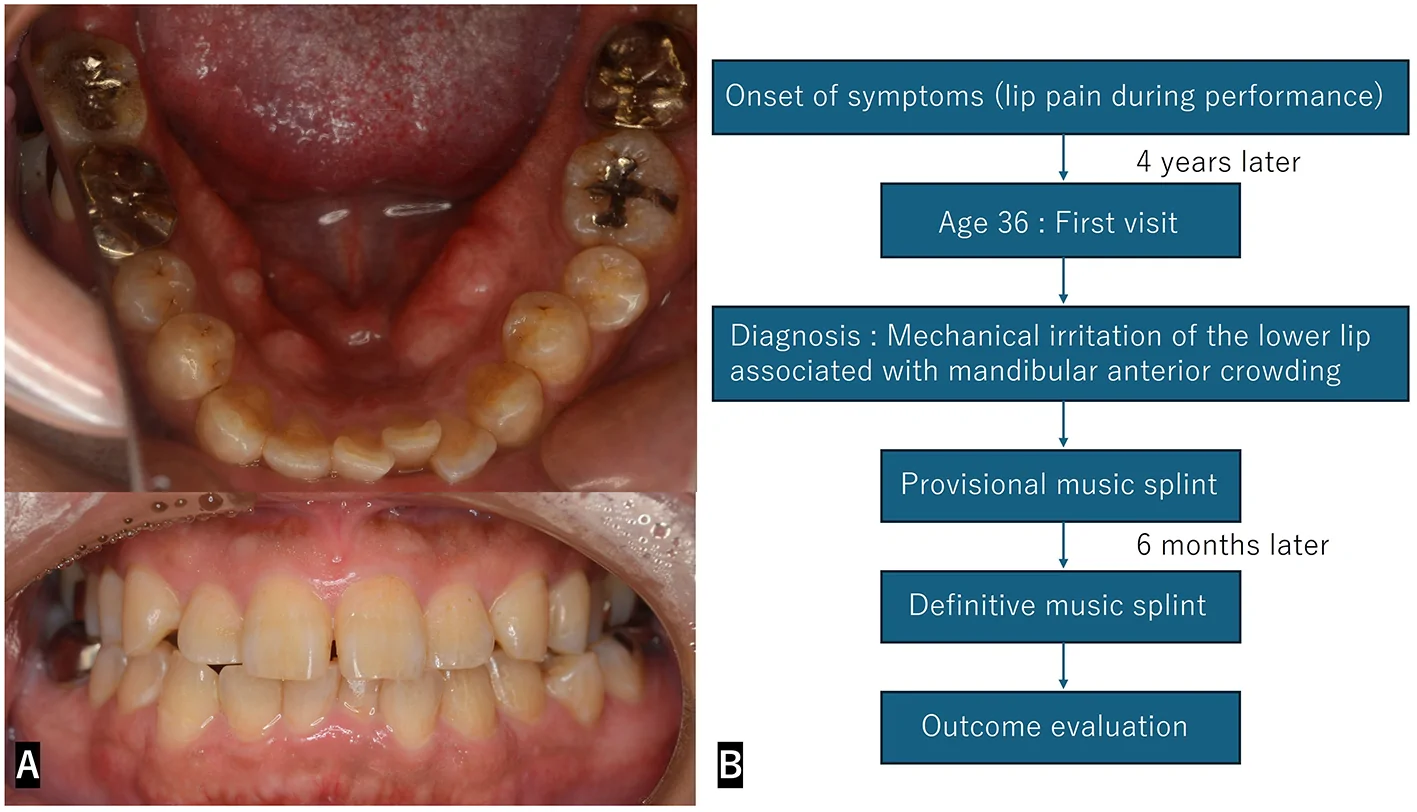
**(A)** Intraoral condition of the patient at the first visit. **(B)** Timeline of the clinical course.

Based on these findings, mechanical irritation of the lower lip caused by contact between the mouthpiece and the mandibular anterior teeth was considered a contributing factor to the patient's symptoms. However, other dental factors, including surface irregularities or wear of the incisal edges, could not be completely excluded as potential contributors to the lip irritation. Because irreversible modification of the dentition could potentially affect the patient's embouchure and playing sensation, a conservative and reversible approach was prioritized. To reduce direct mechanical pressure between the teeth and the lip while playing, music splint therapy was planned. The timeline of the clinical course and intervention in this case is shown in Figure 1B.

### 2.2 Fabrication of the provisional music splint

A provisional music splint was fabricated using conventional techniques. Impressions of the maxillary and mandibular arches were made using irreversible hydrocolloid (Hygedent Normal Set; Ci Dental, Tokyo, Japan), and study models were poured in dental stone (New Ci Hard Gypsum Yellow; Ci Dental). A music splint covering the mandibular anterior teeth was then fabricated using a polyethylene terephthalate disk (Splint Clear, 1.5 mm; Yamahachi Dental, Aichi, Japan). The appliance was designed to create a smooth surface between the teeth and the lower lip without significantly altering the occlusal relationship.

The provisional splint was fitted and adjusted chairside using carbide burs, and further adjustments were made using dental acrylic resin (Unifast III, GC Corporation, Tokyo, Japan). During trial playing with the appliance, the patient was asked to provide feedback regarding comfort, stability, and playing sensation. Based on this feedback, minor adjustments were made to the external and intaglio surfaces of the splint. Because the patient was a trombone player and the mouthpiece was applied against the lips during performance, particular attention was paid to creating a smooth and relatively flat labial surface. The surface of the splint was subsequently polished to ensure smoothness (Figure 2A).

**Figure 2:**
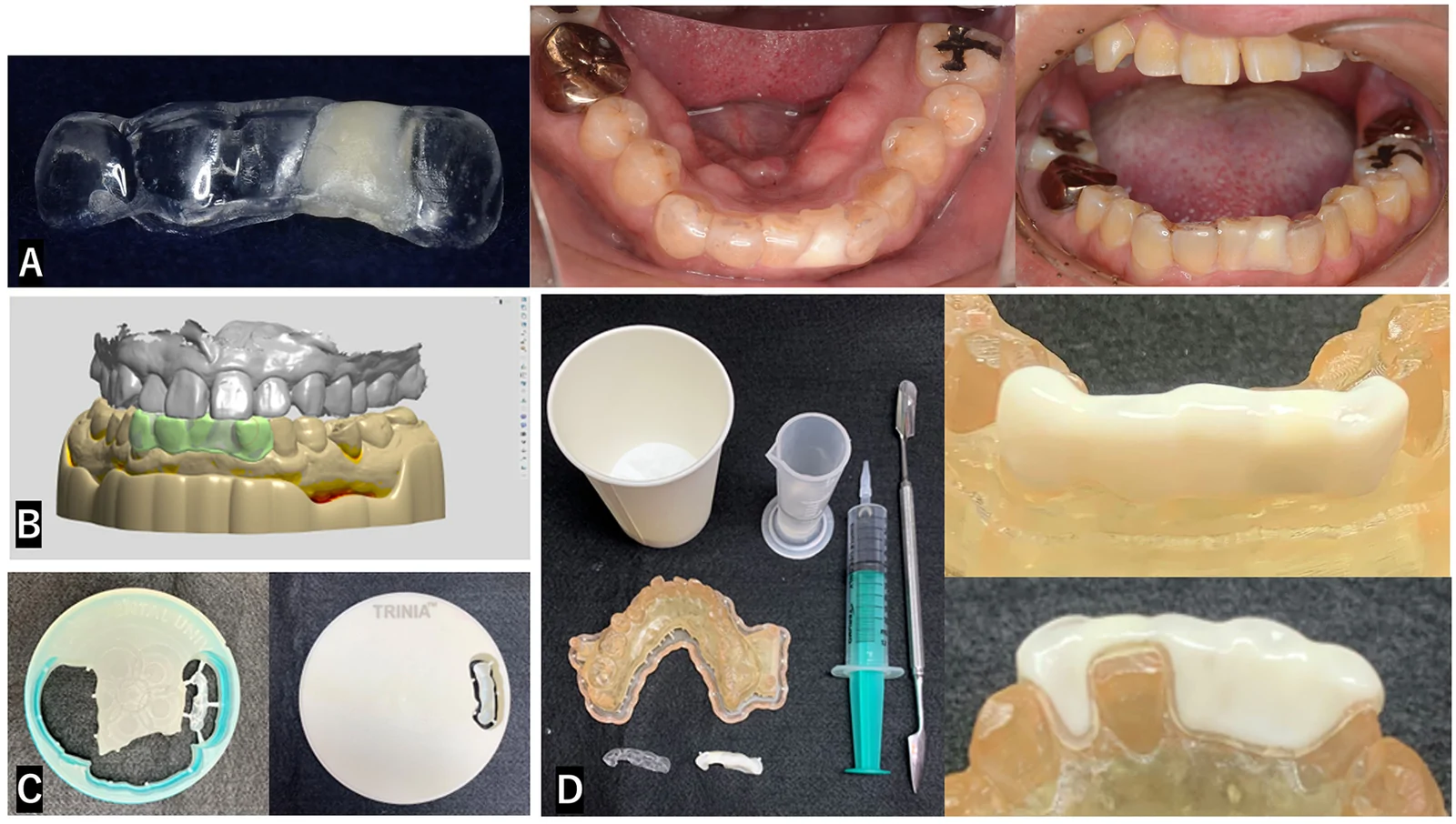
**(A)** Provisional music splint and intraoral views with the splint in place. **(B)** Digital design of the music splint using dental computer-aided design software. **(C)** The parts of the music splint milled from two different material disks. **(D)** Assembling of the music splint and labial and lingual views of the assembled splint on a 3D-printed jaw model.

After using the provisional music splint, the patient reported a reduction in lip pain, which was his primary complaint, along with an improvement in playing comfort. In addition, he noted that his sound quality was positively evaluated by others during performance. Based on these favorable results, the patient expressed a desire to further optimize the appliance.

### 2.3 Digital workflow and fabrication of the definitive music splint

Six months after delivery of the provisional appliance, a definitive music splint was fabricated using a digital workflow. The provisional splint was scanned with a model scanner (D2000; 3Shape, Copenhagen, Denmark) to capture its external morphology. In addition, digital impressions of the dental arches were made using an intraoral scanner (TRIOS 3; 3Shape). As shown in Figure 2B, the definitive music splint was designed using dental computer-aided design software (Dental System; 3Shape).

The definitive music splint extended from the mandibular right canine to the left lateral incisor and had a scalloped margin. At the mandibular left central incisor, the splint covered the labial surface without lingual coverage. The inner component had a uniform thickness of 0.4 mm, with an average thickness of approximately 1.0 mm. As shown in Figure 2C, the appliance components were milled from a disk fabricated using auto-polymerizing denture base acrylic resin (Palapress vario, Kulzer, Hanau, Germany) and a fiber-reinforced composite resin disk (Trinia; Shofu, Tokyo, Japan). Initially, fabrication using the acrylic resin alone was attempted; however, fracture occurred during fabrication at a thin area resulting from the relationship with the opposing dentition. Therefore, the fiber-reinforced composite resin was incorporated to reinforce the thin area. As shown in Figure 2D, the two components were assembled using autopolymerizing denture base acrylic resin (Palapress Vario; Kulzer). The definitive music splint was delivered and evaluated during trial playing. Because its morphology was reproduced from the previously adjusted provisional splint using the digital workflow, little additional adjustment was required. Areas requiring adjustment were identified primarily based on the patient's feedback regarding lip and intraoral discomfort, splint stability, and playing sensation, together with clinical inspection. The labial, lingual, and intaglio surfaces were evaluated in relation to patient comfort and splint stability. Minor adjustments were performed using a silicon carbide bur, followed by polishing with a silicone point to obtain a smooth surface (Figure 3A).

**Figure 3:**
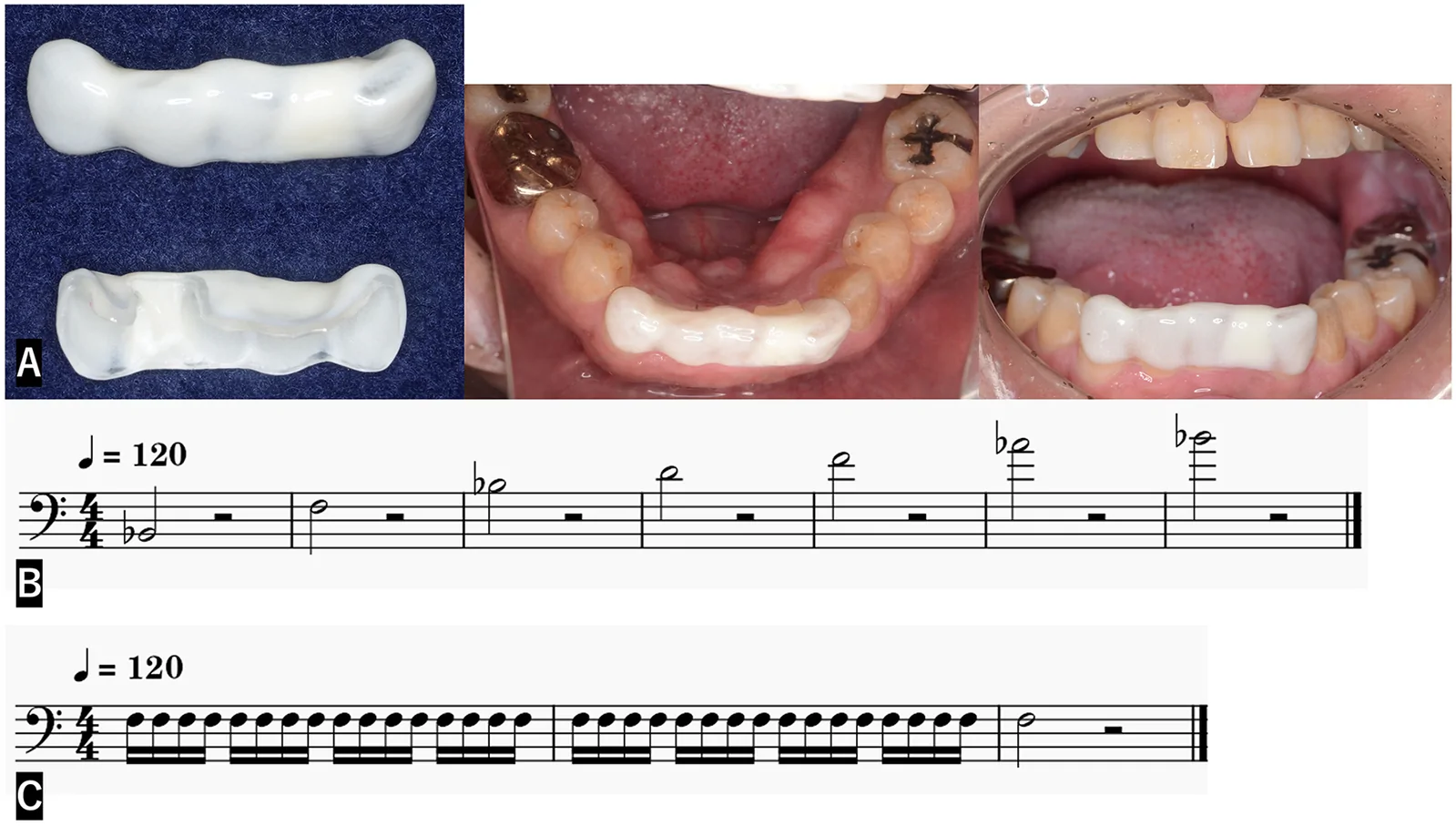
**(A)** Labial and lingual views of the definitive music splint and intraoral views with the splint in place. **(B)** Notes played for the psychoacoustic analysis. **(C)** Notes played for the rhythm analysis.

### 2.4 Outcome measures

The effectiveness of the music splint was evaluated using both subjective and objective measures. Subjective assessment was performed using a visual analog scale (Hattori et al., 2026). The patient rated lip pain, discomfort of the music splint, stability of the music splint, ease of splint contact, ease of playing, fatigue, performance sound, and willingness to use the music splint. The patient retrospectively rated each condition based on his recollection of the playing experience. Performance quality was objectively evaluated using psychoacoustic analysis (Hattori et al., 2015). Recordings were made under four conditions: without a music splint, with the provisional music splint, with the definitive music splint, and with the definitive music splint after adjustment. All recordings were performed on the same day in a soundproof room. The test sounds were recorded in the order of the definitive music splint, without a music splint, provisional music splint, and adjusted definitive music splint. A microphone (LA-5120; Ono Sokki, Kanagawa, Japan) and a sound interface (Sound Blaster Extigy; Creative Technology Ltd., Singapore) were used for recording, and the signals were analyzed using acoustic analysis software (Oscope 2; Ono Sokki). The microphone was positioned 30 cm from the bell of the trombone, and the recording level during ff playing was confirmed to remain within the 30–120 dB measurement range without overload.

As part of the dynamic analysis, the patient was asked to perform specified notes (Figure 3B) at three dynamic levels (pianissimo [pp; very soft], mezzo forte [mf; moderately strong], and fortissimo [ff; very strong]). Each specified note at each dynamic level was recorded once. Each note was sustained as a half note, and loudness and sharpness values over the sustained portion were averaged for each note. For each note, two psychoacoustic parameters, loudness (Zwicker et al., 1957) and sharpness (von Bismarck, 1974), were measured. In addition, rhythm analysis was conducted to evaluate the stability of note duration between repeated notes (Figure 3C) (Hattori et al., 2023). These analyses were used to assess potential changes in performance characteristics associated with use of the splint.

## 3 Results

The definitive music splint fit well. The patient did not feel tooth discomfort or pain when the splint was worn. The patient could easily place and remove the music splint and he could clean it by himself. No adverse or unanticipated events were observed.

### 3.1 Subjective evaluation

The results of subjective assessment using the visual analog scale demonstrated improvement in lip pain and playing comfort when the patient used the provisional and the definitive music splints (Figure 4A). Compared with the condition without a splint, both types of splints were associated with reduced lip pain and improved ease of performance. The definitive splint received the most favorable overall ratings, including stability, ease of contact, ease of playing, reduced fatigue, perceived sound quality, and willingness to use the device during performance.

**Figure 4:**
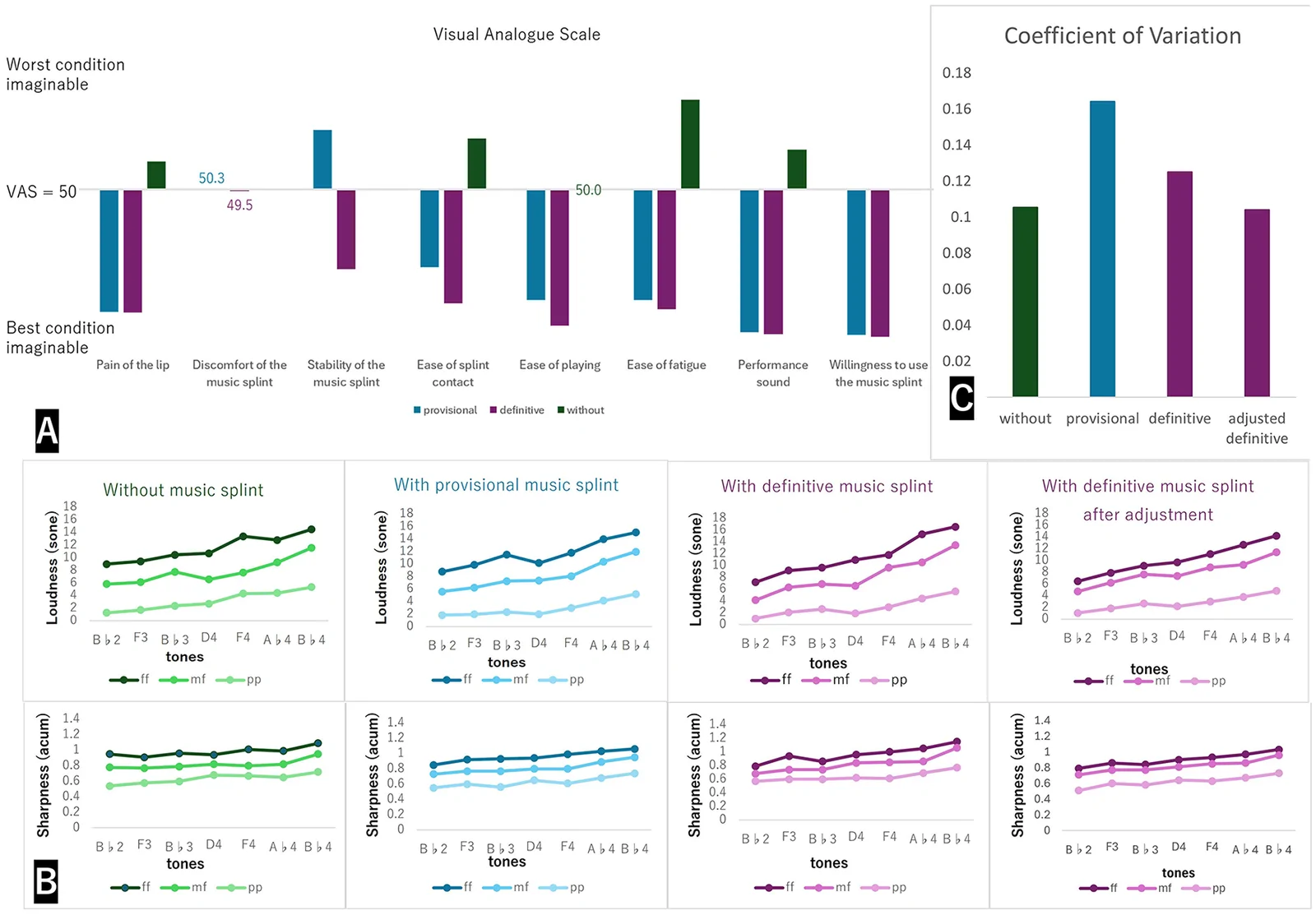
**(A)** Results of subjective analysis using a visual analog scale. **(B)** Results of the psychoacoustic analysis. **(C)** Results of the rhythm analysis.

The patient also reported that he was able to practice for longer periods without discomfort when wearing each of the music splints compared with playing without a splint.

### 3.2 Psychoacoustic analysis

The psychoacoustic analysis results showed that loudness values increased consistently with dynamic level (pp <mf <ff) under all experimental conditions (Figure 4B). Sharpness also generally increased with dynamic level across the four conditions. These findings indicate that the expected dynamic patterns of the measured psychoacoustic parameters were maintained across the four conditions. The use of either music splint did not produce marked deterioration in loudness or sharpness compared with the no-splint condition.

### 3.3 Rhythm analysis

For assessing rhythm stability, the coefficient of variation in note duration is shown in Figure 4C. The provisional splint showed slightly higher variability compared with the no-splint condition. However, after adjustment of the definitive splint, the coefficient of variation decreased to a level similar to that observed without a splint.

## 4 Discussion

In the present case, the use of a music splint by a professional trombonist was associated with a reduction in lip discomfort and pain, as well as improvements in playing comfort and performance endurance. In addition, psychoacoustic and rhythm analyses demonstrated that, when the adjusted definitive music splint was used, performance characteristics were comparable to those observed without a splint. These findings suggest that the appliance alleviated lip symptoms without adversely affecting playing quality.

Playing a wind instrument requires the coordinated functioning of the orofacial region to form and maintain the embouchure. In this case, crowding of the mandibular anterior teeth was observed, and the patient reported irritation in the same region of the lower lip during prolonged playing. These findings suggest that localized mechanical irritation of the lower lip may have occurred during trombone performance. This interpretation is consistent with the observations of Herman (1974), who described that mouthpiece pressure during brass instrument playing can compress the lips against the anterior teeth. Although other dental factors, including surface irregularities or wear of the incisal edges, could also have contributed to the lip irritation, irreversible interventions such as tooth reshaping were not initially performed. Because even minor alterations to the dentition may affect the embouchure of professional musicians, we prioritized a conservative and reversible approach using a removable music splint.

The results of the visual analog scale showed the use of a music splint reduced lip discomfort and improved performance endurance. These findings are consistent with a report by van der Weijden et al. (2018), which suggested that tooth position and occlusal conditions may influence embouchure comfort and playing ability. In addition, previous research by Hattori et al. (2014) demonstrated that changes in oral morphology can influence acoustic characteristics of wind instrument performance, supporting the possibility that modifying the oral environment may affect musical function.

Lip pain and inflammation are important clinical problems among wind instrument players because they can affect both playing quality and endurance. In the present case, the patient experienced discomfort and pain in the lower lip during prolonged playing. This observation is consistent with the findings of Storms et al. (2016), who reported the prevalence of embouchure-related disorders among brass musicians and suggested that prolonged mechanical loading on the lips may contribute to symptom development.

Music splint therapy, which interposes a smooth surface between the teeth and the lip, may represent an effective therapeutic approach for such problems. In the present case, the reduction in lip pain and improvement in playing comfort are consistent with previous findings that splint characteristics influence pain reduction and playing sensation (Nii et al., 2023). Similarly, the improvement in performance observed in this case aligns with reports demonstrating the beneficial effects of splint therapy on musical function (Mitchell et al., 2021).

In the present case, psychoacoustic and rhythm analyses were performed to objectively evaluate musical performance. The results showed no marked deterioration in loudness, sharpness, or rhythm stability when the adjusted definitive music splint was used. These findings are consistent with previous reports by Tanabe et al. (2022) and Hattori et al. (2023)), which demonstrated that intraoral appliances or prosthodontic interventions had minimal adverse effects on the acoustic characteristics of musical performances.

Furthermore, rhythm variability was lower with the definitive splint after adjustment than before adjustment and was similar to that observed without a splint. However, because only a single measurement was obtained under each condition, this change cannot be attributed solely to the adjustment itself. These observations suggest that careful evaluation during actual performance and minor refinement based on patient feedback may be important when fitting a music splint.

Several limitations of this case should be acknowledged. This report describes a single patient, lacks long-term follow-up, and relies primarily on subjective assessments and a limited set of acoustic parameters. In addition, as each note in difference conditions was recorded once, between-take variability and measurement reproducibility could not be assessed. Further studies involving larger cohorts, different types of wind instruments, and longer observation periods are required to clarify the broader applicability of music splint therapy.

Despite these limitations, this case suggests that a digitally fabricated music splint may reduce lip irritation while preserving musical performance in professional brass players. These findings highlight the potential clinical relevance of music splint therapy in the emerging field of music dentistry.

## 5 Patient perspective

The patient reported that prolonged playing caused pain in the lower lip, which made it difficult to continue performing. After using the music splint, the pain was reduced, and discomfort during performance decreased. The patient also noted that longer practice sessions became possible and that anxiety related to performance was alleviated. Furthermore, the patient perceived improvements in sound stability and ease of playing.

## 6 Conclusion

In this case, use of a music splint was associated with reduced lip discomfort and improved playing endurance in a professional trombonist with mandibular anterior crowding. Importantly, psychoacoustic and rhythm analyses indicated that the adjusted definitive splint preserved key performance characteristics without adversely affecting sound quality. These findings suggest that music splint therapy may be a useful clinical approach for managing lip irritation in wind instrument players while maintaining their level of musical performance. The integration of digital workflows may further facilitate the precise design and fabrication of such appliances. Future studies involving larger cohorts and longer follow-up are needed to clarify the broader applicability of this approach.

## Data Availability

The data underlying the findings of this study are not publicly available owing to privacy concerns but will be made available by the corresponding author upon reasonable request.
